# Survival outcomes among patients with multiple myeloma in the era of novel agents: exploratory assessment using an electronic medical record database in Japan

**DOI:** 10.1371/journal.pone.0285947

**Published:** 2023-05-31

**Authors:** Shuji Uno, Shuichi Midorikawa, Kei Inoue, Daisuke Ichikawa, Tomoki Ito, Junya Kuroda, Kenshi Suzuki

**Affiliations:** 1 Japan Medical-Hematology, Bristol-Myers Squibb K.K., Tokyo, Japan; 2 Biometrics and Data Sciences, Bristol-Myers Squibb K.K., Tokyo, Japan; 3 SUSMED, Inc, Tokyo, Japan; 4 First Department of Internal Medicine, Kansai Medical University, Osaka, Japan; 5 Division of Hematology and Oncology, Kyoto Prefectural University of Medicine, Graduate School of Medical Science, Kyoto, Japan; 6 Department of Hematology, Japan Red Cross Medical Center, Tokyo, Japan; Keio University School of Medicine, JAPAN

## Abstract

Despite recent advances in the range of therapies available for the treatment of multiple myeloma (MM), there are limited data surrounding survival outcomes and baseline characteristics influencing survival in general clinical practice in Japan. The aim of this study was to use electronic medical records (EMRs) to examine overall survival (OS) and prognostic factors in Japanese patients with MM. We extracted EMRs in the Real World Data (RWD) database of patients with a confirmed diagnosis of MM and treatment history with bortezomib, thalidomide, and/or lenalidomide. OS and prognostic factors for OS were analyzed using a univariate analysis and decision tree model. Of the 6509 patients in the database with a diagnosis of MM, 1565 were eligible. Patients had a median (range) age of 72 (23–92) years, a median OS of 53.5 months, and a 5-year OS rate of 45.6%. In alignment with previous studies, International Staging System stage and age were prognostic of OS. In addition, platelet and erythrocyte counts, chloride, total protein, C-reactive protein, and lactate dehydrogenase levels were identified as important prognostic factors for OS and were used to pilot a simple prognostic tool. In conclusion, we found that the survival outcomes extracted from EMRs in the RWD of Japanese patients with MM aligned with a previous retrospective study from Japan. Baseline laboratory parameters prognostic for OS were explored with additional factors to International Staging System and age identified. These might be used to optimize treatment selection, although further investigation using additional data sources is required.

## Introduction

Multiple myeloma (MM) is a hematologic neoplasm characterized by proliferation of plasma cells in the bone marrow. In Japan, MM has a yearly incidence of around 5 new cases per 100,000 people and results in approximately 4000 deaths per year.^1^ Generally, treatment for MM is only initiated when the disease progresses from a precancerous, asymptomatic condition (such as monoclonal gammopathy of undetermined significance or smoldering MM) to symptomatic MM [1, 2].

Many new therapies have been developed for the treatment of MM since the early 2000s [3]. In Japan, the first 3 new drugs approved for the treatment of MM were bortezomib (BORT) in December 2006, thalidomide (THAL) in October 2008, and lenalidomide (LEN) in June 2010. Since then, the immunomodulatory drug pomalidomide, the proteasome inhibitors carfilzomib and ixazomib, and the monoclonal antibodies elotuzumab, daratumumab, and isatuximab have been approved in succession. The introduction of these agents has improved outcomes for patients with MM [4, 5]; the International Myeloma Working Group (IMWG) reported a median overall survival (OS) of 44 months in 2005 [6], the Mayo Clinic reported a median OS of 62.4 months in 2014 [4], and the Japanese Society of Myeloma reported a median OS of 60.6 months in 2015 [7]. However, MM remains an incurable disease, and survival outcome depends on the individual patient’s demographic and clinical characteristics and treatment regimen(s) [4, 6, 7].

To understand survival outcomes of patients with MM, various reports have sought to identify prognostic factors. In 1975, the Durie/Salmon system was proposed as a method of MM clinical staging in which myeloma cell mass was calculated based on assessments of hemoglobin, calcium, M-protein production, urine light chain and serum creatinine levels, and bone lesions on X-ray [8]. Serum β_2_-microglobulin was later reported to be an important prognostic factor and was integrated into several prognostic models in the 1980s [9–12]. In 2005 the IMWG developed the International Staging System (ISS) that uses serum β_2_-microglobulin and albumin levels to stratify patients with MM into prognostic risk groups [6]; this system is widely used in daily clinical practice. In 2015 lactate dehydrogenase (LDH) levels and cytogenetic abnormalities were incorporated into the ISS to develop the revised ISS (R-ISS) [13], and these prognostic indices are used for predicting outcomes in Japanese patients with MM [7, 14]. Recently, the Connect^®^ MM registry in the USA reported that advanced age, no deletion of the short arm of chromosome 17, no history of triplet therapy, decreased mobility (dimension of the EuroQol-5), higher ISS stage, presence of solitary plasmacytoma, a history of diabetes, lower platelet count, higher Eastern Cooperative Oncology Group performance status, and higher serum creatinine levels were all negatively associated with OS in patients with MM [15].

To date, a variety of approaches—including chart reviews and questionnaires—have been used to understand real-world treatment outcomes for patients with MM. Electronic medical records (EMRs) have become available in several commercial databases and are already being used to understand clinical outcomes in patients with MM [16, 17]. In addition, there are now several commercially available databases in Japan [18].

So far, studies analyzing survival outcomes in Japanese patients with MM have been based on questionnaire data collected prior to 2012 [7, 14]. Using commercially available databases in Japan to analyze prognostic factors and evaluate the prognosis of patients with MM could facilitate understanding of survival outcomes, as well as contribute to the design of clinical studies and the planning of treatment strategies in daily clinical practice. However, the majority of these databases contain information from only 1 or 2 sources (EMRs, claims, laboratory test results, or Diagnosis Procedure Combination [DPC] records). To examine survival outcomes thoroughly requires that there is no age bias in the data, survival information is not limited to hospital mortality, and as much laboratory data are available as possible. The integrated Real World Data (RWD) database contains EMRs, laboratory test results, claims, and DPC records [19, 20], and therefore meets these requirements. However, to our knowledge, no studies of survival in patients with MM have used this database to date.

The aim of this study was to examine survival outcomes among patients with MM using the RWD database and to explore prognostic factors affecting OS.

## Methods

### Study design and cohort construction

This was an observational retrospective study using the RWD database, which is maintained by the Health, Clinic, and Education Information Evaluation Institute (Kyoto, Japan) with support from Real World Data Co., Ltd (Kyoto, Japan). The RWD database contains data from approximately 190 institutions, including 91 acute care hospitals with the DPC per-diem payment system. Because the study used anonymized data, institutional ethics approval and informed consent were not required in accordance with ethical guidelines in Japan. The study protocol was reviewed by Bristol Myers Squibb internal review board in terms of scientific validity and materiality.

For inclusion in the analysis, data were identified from patients with a confirmed diagnosis of MM and with a history of ≥ 1 prior treatment with BORT, THAL, or LEN. Because symptomatic and asymptomatic MM were not recorded as separate diseases in the database, treatment history was among the inclusion criteria to select patients with symptomatic MM. In Japan, MM drugs other than BORT/LEN/THAL are often used as salvage therapy after relapse or are used in combination with them [1]. Therefore, it was considered that MM patients could be rationally selected by having a history of treatment with at least one of these three drugs.

**Fig 1:**
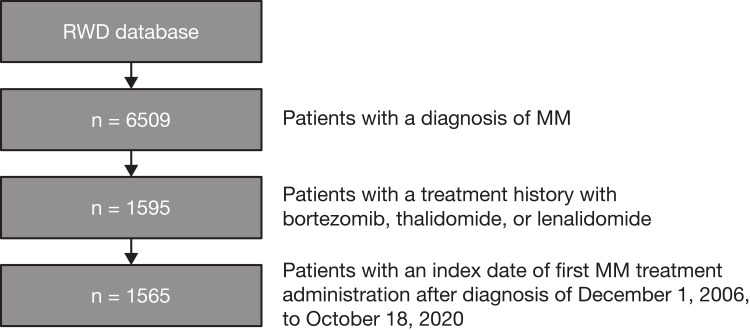
Flow diagram of patient selection. Abbreviations: MM = multiple myeloma; RWD = Real World Data.

The date of administration of the first MM treatment after diagnosis was defined as the index date, and only patients whose index date was December 1, 2006, to October 18, 2020, were included in the analysis to minimize selection bias toward patients who survived to the earliest approval date among the 3 drugs (BORT, December 1, 2006). Patient outcomes were analyzed during the follow-up period: index date to date of death, latest date in the database, or lost to follow-up (whichever occurred first). The patient selection overview is shown in **Fig 1**.

The primary objective of the analysis was to describe the current real-world OS rate of patients with symptomatic MM using the RWD database. Secondary objectives included describing the baseline demographics and clinical characteristics of patients and examining prognostic factors associated with OS.

### Statistical approach

Descriptive statistics were used to describe patient demographic and baseline clinical characteristics. Time-to-event data were analyzed using the Kaplan–Meier method and provided the median and 95% confidence interval (CI; if available), 25th percentile, and 75th percentile. Patients who did not experience an event were censored, with duration period being the time between index date and end of study/lost to follow-up (whichever occurred first).

The RWD database contained the results of blood tests and urinalysis data collected in daily clinical practice. Variables analyzed included “first MM regimen (BORT, LEN, BORT+LEN, and Other)”, “sex”, “age”, “laboratory test results with < 50% missing data”, and “prescription history for treatment used by more than 10% of patients during baseline period”. Continuous variables were log-transformed and normalized (variance, 1; mean, 0). To mitigate the effect of missing data, multiple imputation methods were applied using a fully conditional specification regression method [21]. Baseline characteristics were collected on and 30 days (180 days for the history of drug prescription) prior to the index date.

For variable selection methods, both forward stepwise and backward stepwise were examined, with backward stepwise selected due to superior accuracy [22, 23]. Categorical variables of ≥ 3 classes were converted to binary variables, and those that were not significant were excluded.

A Cox proportional hazards model and a random survival forest (RSF) model were used to assess factors influencing OS. A log-rank test was performed using the threshold at which the absolute value of the log-rank test statistic was the highest [24]. Survival outcomes were stratified using a decision tree.

## Results

### Patient cohort identification

A total of 6509 patients with a confirmed diagnosis of MM were identified from the RWD database. Among these patients, 4914 patients had no history of treatment with BORT, LEN, or THAL. Therefore, these patients may have been asymptomatic MGUS or SMM and were excluded from this study. After excluding patients who started MM treatment before December 1, 2006, or after October 18, 2020, 1565 patients remained in the study (**Fig 1**). Among these patients, the median (range) follow-up was 25.4 months (0.0–155.9).

### Patient characteristics

Of the 1565 patients included in the analysis, 759 patients (48%) were female. The median (range) age was 72 years (23–92), with 662 patients (42%) aged > 74 years and 21%, 43%, and 36% of patients, respectively, with ISS stages I, II, and III. A total of 1170 patients (75%) received BORT, LEN, or both as their first treatment regimen (**Table 1**). Sixty-one baseline variables, including blood test and urinalysis data and the number of patients included in each analysis, are shown in **S1 Table**.

**Table 1:** Baseline characteristics.

Characteristic	N = 1565
Sex, n (%)	
Female	759 (48)
Male	806 (52)
Median (range) age, years	72 (23–92)
Age, years	
< 65	343 (22)
65–74	560 (36)
> 74	662 (42)
ISS Stage, n (%)	
I	223 (21)^a^
II	449 (43) ^a^
III	379 (36) ^a^
Missing	514 (33)
First regimen, n (%)	
Bortezomib	642 (41)
Bortezomib + lenalidomide	53 (3)
Lenalidomide	475 (30)
Other	395 (25)

^a^Percentages shown are per the 1051 patients with ISS stage available.

ISS = International Staging System.

### Overall survival

**Fig 2:**
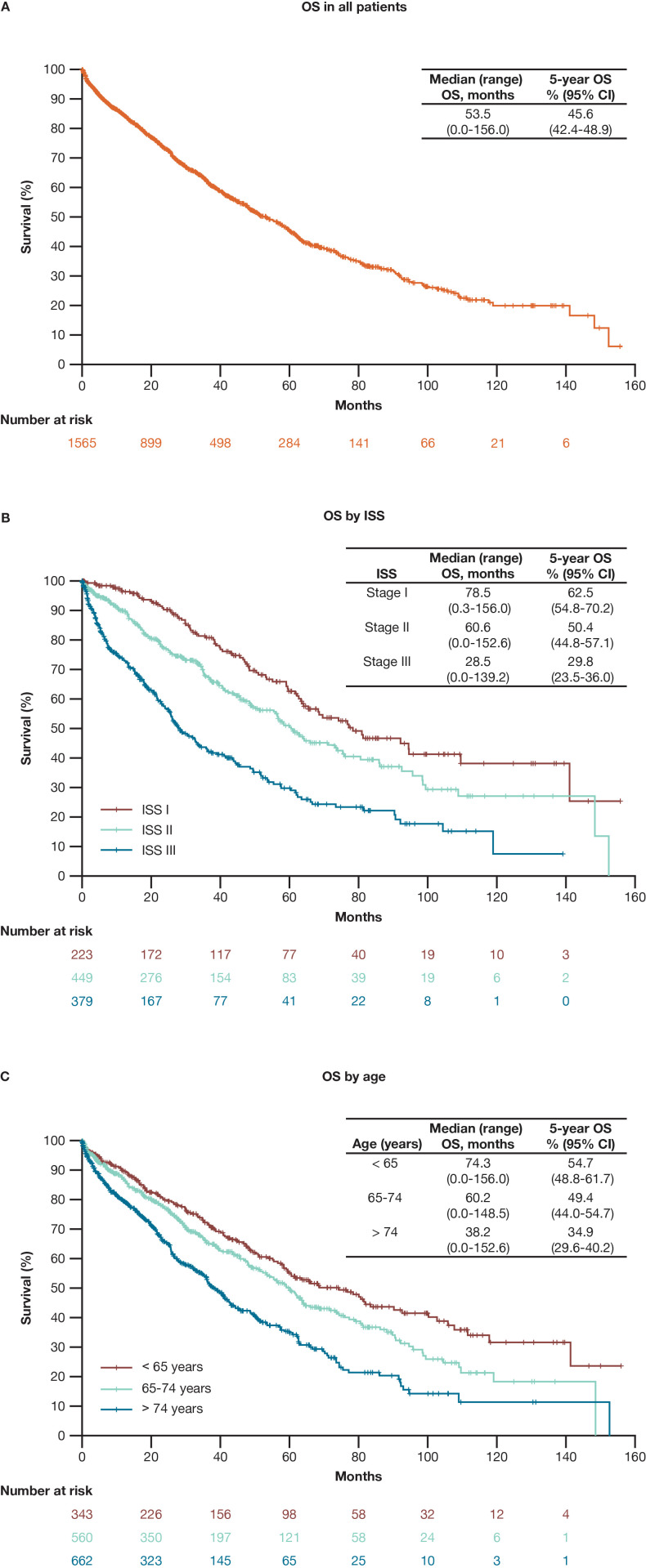
OS in patients with multiple myeloma. Abbreviations: CI = confidence interval; ISS = International Staging System; OS = overall survival.

Of the 1565 patients analyzed, 688 patients (44%) died. Overall, the median (range) OS was 53.5 (0.0–156.0) months and the 5-year OS rate was 45.6% (95% CI, 42.4–48.9) (**Fig 2A**). Patients aged < 65 years and those with ISS stage I MM had higher 5-year OS rates and median OS than patients aged ≥ 65 years and those with ISS stage II or III MM (**Fig 2B** and **2C**).

### OS prediction model and factors influencing OS

A total of 61 baseline variables (**S1 Table**) were entered into the 2 multivariable models (Cox [**S2 Table**] and RSF [**S3 Table**]). In addition to age and ISS stage, which are already used as standard prognostic factors for MM, 6 variables (platelet and erythrocyte count; chloride concentration; and total protein in blood [TP], C-reactive protein, and LDH levels) were identified as prognostic factors for OS in both models (**Table 2**). Both the Cox and RSF models showed similar predictive accuracy (Cox: cross-validation in training data [CV-train], C-index = 0.72; cross-validation in test data [CV-test], C-index = 0.69. RSF: CV-train, C-index = 0.68; CV-test, C-index = 0.69). Using this method, rebamipide use at baseline was identified as a significant factor in the prognosis of OS in Japanese patients with MM in the Cox model (**Table 2**).

**Table 2:** Baseline variables associated with OS.

**Cox proportional hazards model**				
**Variable**	**Variable group**	**Coefficient**	**HR (95% CI)**	***P* value**
Rebamipide treatment	Drug	-0.345	0.708 (0.553–0.908)	0.006
Albumin, g/dL	Blood	-0.231	0.794 (0.719–0.876)	0.000
**Platelet count, 10** ^**4**^ **/μL**	**Blood**	**-0.209**	**0.811 (0.748**–**0.880)**	**0.000**
**Erythrocyte count, 10** ^**4**^ **/μL**	**Blood**	**-0.188**	**0.828 (0.735**–**0.934)**	**0.002**
**Chloride, mEq/L**	**Blood**	**-0.180**	**0.835 (0.765**–**0.913)**	**0.000**
**Total protein, g/dL**	**Blood**	**-0.166**	**0.847 (0.777**–**0.924)**	**0.000**
Creatinine, mg/dL	Blood	-0.140	0.869 (0.781–0.967)	0.010
Aspartate aminotransferase, U/L	Blood	-0.131	0.877 (0.804–0.957)	0.003
Eosinophil, U/L	Blood	-0.097	0.907 (0.834–0.987)	0.024
**C-reactive protein, mg/dL**	**Blood**	**0.086**	**1.090 (1.003**–**1.184)**	**0.042**
Calcium, mg/dL	Blood	0.159	1.172 (1.065–1.290)	0.001
Male	Demographic	0.196	1.216 (1.030–1.437)	0.021
Age	Demographic	0.264	1.302 (1.196–1.418)	0.000
**Lactate dehydrogenase, U/L**	**Blood**	**0.408**	**1.503 (1.354**–**1.669)**	**0.000**
ISS stage III	Demographic	0.421	1.524 (1.188–1.955)	0.001
**Random survival forest model**				
**Variable**	**Variable group**		**Permutation importance**	
**Lactate dehydrogenase, U/L**	**Blood**		**0.0053**	
β_2_-microglobulin, mg/dL	Blood		0.0051	
**Platelet count, 10** ^**4**^ **/μL**	**Blood**		**0.0039**	
ISS stage	Demographic		0.0033	
Age	Demographic		0.0029	
**Erythrocyte count, 10** ^**4**^ **/μL**	**Blood**		**0.0028**	
Albumin, g/dL	Blood		0.0026	
Hemoglobin, g/dL	Blood		0.0025	
**Chloride, mEq/L**	**Blood**		**0.0020**	
Blood urea nitrogen, mg/dL	Blood		0.0020	
Neutrophil lymphocyte ratio	Blood		0.0018	
Age group^a^	Demographic		0.0017	
Cholinesterase, U/L	Blood		0.0016	
**Total protein, g/dL**	**Blood**		**0.0015**	
**C-reactive protein, mg/dL**	**Blood**		**0.0014**	

Rows in **bold** indicate prognostic factors for OS identified by both models.

Abbreviations: CI = confidence interval; HR = hazard ratio.

^a^Patients were grouped by age (< 65, 65–74, and > 74 years), and this was used as a variable.

### Univariate analysis to identify factors of interest

**Fig 3:**
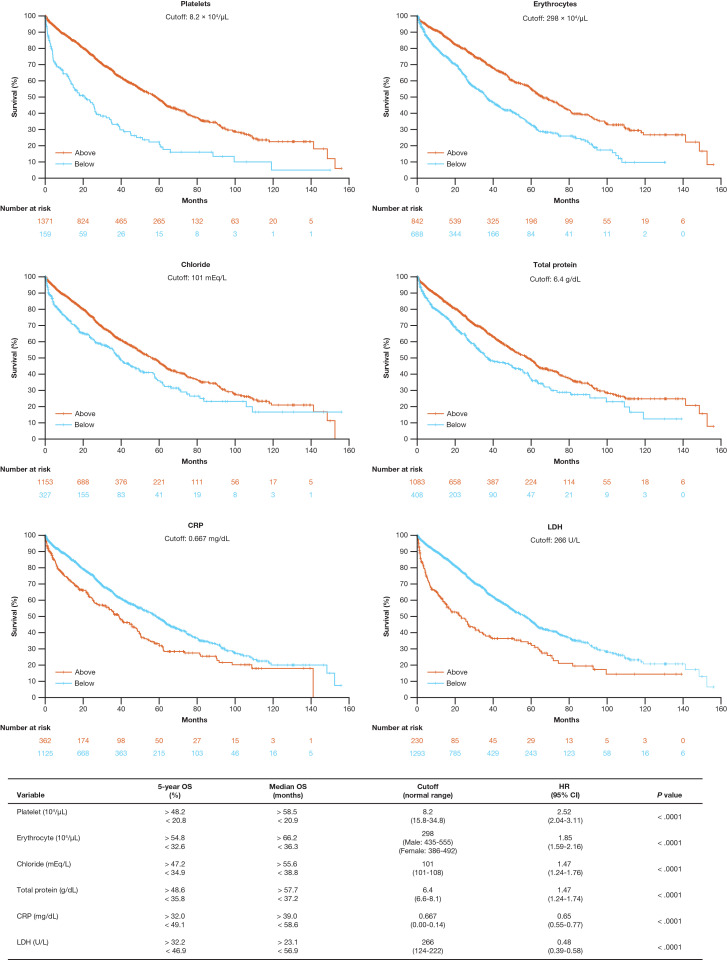
Univariate analysis of baseline characteristics of interest. Kaplan–Meier and log-rank: The threshold at which the absolute value of the log-rank test statistic reaches the maximum (minimum *P* value) and the corresponding HR and *P* value for factors considered important other than ISS, albumin, β_2_-microglobulin, and age. Normal range shown in the figure is the value published by the Laboratory Department of the National Cancer Center Hospital in Japan [32, 33]. Abbreviations: CI = confidence interval; CO = cutoff; CRP = C-reactive protein; HR = hazard ratio; ISS = International Staging System; LDH = lactate dehydrogenase; OS = overall survival; TP = total protein.

Next, the threshold values of baseline blood and urine factors identified as prognostic were analyzed to determine at which the absolute value of the log-rank statistic reached its maximum was estimated, and data were stratified above or below the threshold to examine the difference in OS (**Fig 3** and **S4 Table**). Higher platelet, erythrocyte, chloride, and TP levels and lower C-reactive protein and LDH levels were positively associated with OS.

### Construction of a simple prognostic classification system

Because the results of this study support the use of the ISS as a prognostic tool, the 6 additional variables identified as prognostic factors for OS in the Cox and RFS models were added to a decision tree to determine whether a simple prognostic classification system could be constructed using these variables. Threshold values of platelet, TP, chloride, and LDH levels were all identified as potentially useful for simple prognostic classification (**S1 Fig**) in addition to the commonly used β_2_-microglobulin levels and/or age, with a predictive accuracy comparable to ISS (**S5 Table**).

## Discussion

To our knowledge, this is the first study to use the RWD database to evaluate survival outcomes and explore prognostic factors affecting OS in patients with MM in the era of novel agents in Japan. A total of 1565 patients (median [range] OS, 53.5 [0.0–156.0] months; 5-year OS rate, 46%) fulfilled the eligibility criteria and were included in the analysis.

The median (range) age of the patients with MM included in the analysis was 72 (23–92) years, and the proportion of patients aged > 74 years was slightly higher than that of previous reports in Japan [7]. This may be due to the patient population at the institutions included in the RWD database, and the age distribution may influence the estimation of overall prognosis. However, there should be no major differences in sex or ISS distribution compared with previous reports [7].

The median follow-up period was only 25.4 months, which suggests that a sufficiently long observation period could not be secured. Because the RWD database could not completely exclude the possibility of a history of MM treatment at another hospital before the index date, the median OS may not have been properly estimated. However, 668 deaths (44% of patients) were reported and the median OS was 53.5 months, which is largely consistent with the OS previously reported for patients with MM in Japan [7]. The accepted prognostic factors of ISS and age were also prognostic for OS in this study. Further accumulation of cases in the RWD database and a longer observation period are needed for a more precise estimation, but because the OS rate is in line with previous reports [4, 7], factors associated with OS could be further explored in this study.

Cox and RSF models were used in this analysis and identified ISS stage, age, albumin, and β_2_-microglobulin levels as prognostic factors, all of which are already considered important factors in predicting survival [6, 10, 12]. Both models analyzed the 61 variables and showed similar predictive accuracy. However, the accuracy of the Cox model that used only 2 variables (age and ISS) was comparable with the models with 61 variables, suggesting that age and ISS stage may have contributed significantly to model accuracy. In addition, the accuracy of the Cox model was almost equivalent to that of the RSF model, which does not require the assumption of proportional hazards. As proportional hazards were maintained, the Cox model was considered applicable. The difference in CV-train/-test in the RSF model was small, therefore it was considered unlikely that overfitting occurred.

Using the models, additional factors were identified as being important for determining the prognosis for OS in patients with MM. Previously, the IMWG examined the significance of a platelet count of < 130,000/μL as a prognostic factor; but the number of patients in this study was low, and therefore platelet count was not entered into the ISS. The platelet count was identified as an important variable in this study by both the Cox and RSF models. When the threshold was set at a platelet count of < 82,000/μL, the number of applicable patients was small (10.4%), but the hazard ratio was 2.52 (95% CI, 2.04 to -3.11), suggesting that platelet count should be considered an important factor for determining the prognosis for Japanese patients with MM (**Fig 3**).

LDH levels, which were previously investigated and reported as an important prognostic factor by the IMWG, were not integrated into the ISS [6]. However, LDH levels were incorporated into the R-ISS [13]. Prognostic factors for patients with MM were also investigated in the Connect^®^ MM registry, and platelet count (< 150,000/μL) but not LDH levels (> 300 U/L) was reported to be important [15]. In this study, LDH levels were identified as an important variable, even when the threshold was set at ≥ 266 U/μL and despite the small proportion of patients (15.1%), clearly suggesting the prognostic importance of LDH levels for Japanese patients with MM (**Fig 3**).

The Durie/Salmon staging system uses creatine levels available in clinical practice to assess the prognosis for patients with MM [8]. Although serum creatinine levels correlated with OS in some studies [4, 15], in others they did not [25]. In this study, other variables were determined to have a greater influence on prognosis than serum creatinine. Although there should be no major bias in terms of the age or sex of the patients analyzed in this study, the significance of creatinine as a prognostic factor may require further verification and follow-up.

The simple prognostic tool developed using non-ISS factors and tested using a decision tree model showed that platelet count, LDH, and chloride levels were prognostic factors for OS (**S1 Fig**). This decision tree was particularly accurate at classifying the group with a poor prognosis but not the group with a good prognosis. In the decision tree algorithm, patients are grouped according to the levels of variables that provide the best separation sensitivity for prognosis, and this may be a result of the characteristics of the method (ie, conditions that clearly define a particular patient group are easy to extract, such as the group with a poor prognosis). Conversely, patient groups whose laboratory values are within the normal range and therefore not clearly defined may not be as easily categorized by the algorithm, such as the group with a good prognosis. Verification of this simple prognostic tool using other data sources may lead to more meaningful results in the future.

Rebamipide use at the baseline period was unexpectedly identified as a significant prognostic factor in the Cox model, but not in the RSF model (**Table 2**). In a previous report, rebamipide downregulated survivin and Aurora-B expression in AGS cells (a human gastric adenocarcinoma cell line) and inhibited cell proliferation [26]. Furthermore, Aurora kinase is overexpressed in many human cancer cells [27] and is also highly expressed in patients with MM [28, 29]. In this study, patients with a history of rebamipide use (n = 212) had a significantly higher median (range) OS (79.1 [62.3–100.0] months) than patients without a history of rebamipide use (n = 1353; 50.4 [45.9–56.0] months) (hazard ratio, 0.661; 95% CI, 0.19–0.841; *P* = 0.000761), and this warrants further investigation.

In a recent report from Germany, LEN-based therapy did not affect OS [30]. In this study, the first MM regimen (BORT, LEN, BORT+LEN, and neither BORT nor LEN) was examined as a variable in both the Cox and RFS models to understand if the first regimen affected OS; however, none of the regimens was a significant influencing factor. This would suggest that prognosis may be affected by a patient’s baseline characteristics rather than the initial therapy; however, given that continued treatment has been reported to correlate with a better prognosis [31], it may be necessary to investigate further the effect of treatment regimen on prognosis.

There are several limitations arising from this retrospective study. In Japan, most facilities with hematology departments are hospitals with the diagnosis procedure combination (DPC) per-diem payment system. Although RWD database contains data from approximately 190 institutions (including 91 acute care hospitals with the DPC), there is a total of 1764 hospitals with the DPC system in Japan. The coverage rate is just over 5%, and the possibility of facility selection bias cannot be excluded. Not all hospitals in Japan are included in the RWD database, which may have introduced bias. For instance, the rate of stem cell transplantation in this study was 7.4% (n = 93/1565), which seems low considering that stem cell transplantation is generally performed in Japan for patients aged < 65 years, who represent 22% of the study population. The inclusion criteria of treatment history with BORT, LEN, or THAL was designed to identify patients with newly diagnosed MM; however, any history of MM treatment at another hospital before the index date is unknown. It cannot be ruled out that there was bias in the prognosis for patients who discontinued early during follow-up, meaning the survival outcomes in this study may have been affected. Eastern Cooperative Oncology Group performance status and fluorescence in situ hybridization test results were not available in the RWD database; therefore, the significance of each baseline factor examined in this study may change if these variables are included. In this study, as a commercial database was used, there are limitations regarding data access. If more information regarding the specific data used in our study is needed, please refer to the contents described in “Data Availability and Data Access”. Finally, it should be noted that all results in this study were obtained using only the RWD database and do not warrant generalizability.

## Conclusions

Using data from the general clinic setting present in the RWD database, this study captures the survival outcomes for patients with MM in the era of novel agents in Japan, suggesting that the RWD database may be a useful resource. In addition to ISS and age, other factors that affect OS were identified. Further investigation using additional data sources are needed to optimize treatment selection for patients with MM.

## Supporting information

S1 Table Baseline characteristics. (DOCX)

S2 Table Baseline factors associated with overall survival by Cox proportional hazards model. (DOCX)

S3 Table Baseline factors associated with overall survival by random survival forest model. (DOCX)

S4 Table Univariate analysis of overall survival by continuous variables. (DOCX)

S5 Table Accuracy of each prognostic classification model constructed using a decision tree. (DOCX)

S1 Fig Survival outcomes stratified with the decision tree. (DOCX)

## Abbreviations

MM: multiple myeloma
BORT: bortezomib
LEN: lenalidomide
IMWG: International Myeloma Working Group
OS: overall survival
ISS: International Staging System
LDH: lactate dehydrogenase
R-ISS: Revised International Staging System
EMR: electronic medical record
DPC: diagnosis procedure combination
RWD: real-world data
CI: confidence interval
TP: total protein
CV-train: cross-validation in training data
CV-test: cross-validation in testing data
