# Supplementary material for: Survival outcomes among patients with multiple myeloma in the era of novel agents: exploratory assessment using an electronic medical record database in Japan
Source: PLoS One. 2023 May 31;18(5):e0285947. doi: 10.1371/journal.pone.0285947 (PMC10231788; doi:10.1371/journal.pone.0285947)
Supplement: S1 Table — (DOCX) [file pone.0285947.s001.docx]

### Table S1. Baseline characteristics.

| **Characteristic** | **Number of patients with data available** | **n (%)** |
| --- | --- | --- |
| Age, years | 1565 |  |
| < 65 |  | 343 (22) |
| 65–74 |  | 560 (36) |
| > 74 |  | 662 (42) |
| ISS stage, n (%) | 1051 |  |
| I |  | 223 (21) |
| II |  | 449 (43) |
| III |  | 379 (36) |
| Treatment history, n (%)^a^ |  |  |
| Acetaminophen | 1565 |  |
| 0 |  | 1395 (89) |
| 1 |  | 170 (11) |
| Acyclovir | 1565 |  |
| 0 |  | 1291 (82) |
| 1 |  | 274 (18) |
| Amlodipine besylate | 1565 |  |
| 0 |  | 1370 (88) |
| 1 |  | 195 (12) |
| Aspirin | 1565 |  |
| 0 |  | 1276 (82) |
| 1 |  | 289 (18) |
| Esomeprazole magnesium hydrate | 1565 |  |
| 0 |  | 1338 (85) |
| 1 |  | 227 (15) |
| Famotidine | 1565 |  |
| 0 |  | 1348 (86) |
| 1 |  | 217 (14) |
| Febuxostat | 1565 |  |
| 0 |  | 1313 (84) |
| 1 |  | 252 (16) |
| Fluconazole | 1565 |  |
| 0 |  | 1314 (84) |
| 1 |  | 251 (16) |
| Furosemide | 1565 |  |
| 0 |  | 1367 (87) |
| 1 |  | 198 (13) |
| Isotonic sodium chloride solution | 1565 |  |
| 0 |  | 828 (53) |
| 1 |  | 737 (47) |
| Lansoprazole | 1565 |  |
| 0 |  | 1189 (76) |
| 1 |  | 376 (24) |
| Magnesium oxide | 1565 |  |
| 0 |  | 1181 (75) |
| 1 |  | 384 (25) |
| Rebamipide | 1565 |  |
| 0 |  | 1353 (86) |
| 1 |  | 212 (14) |
| Sennoside | 1565 |  |
| 0 |  | 1371 (88) |
| 1 |  | 194 (12) |
| Sulfamethoxazole/trimethoprim | 1565 |  |
| 0 |  | 1154 (74) |
| 1 |  | 411 (26) |
| Laboratory test data: urine, n (%)^b^ |  |  |
| Glucose (qualitative analysis) | 878 |  |
| 0 |  | 815 (93) |
| 1 |  | 63 (7) |
| Protein (qualitative analysis) | 837 |  |
| 0 |  | 434 (52) |
| 1 |  | 403 (48) |
| Missing |  | 728 |
| Urobilinogen (qualitative analysis) | 1101 |  |
| 0 |  | 1017 (92) |
| 1 |  | 84 (8) |
| Laboratory test data: blood, mean (SD) |  |  |
| Basophil lymphocyte ratio | 1303 | 0.02 (0.05) |
| C-reactive protein, mg/dL | 1487 | 0.86 (1.98) |
| Eosinophil lymphocyte ratio | 1395 | 0.10 (0.15) |
| Monocyte lymphocyte ratio | 1424 | 0.34 (0.37) |
| Neutrophil lymphocyte ratio | 1229 | 3.90 (6.68) |
| Platelet lymphocyte ratio | 1428 | 0.95 (15.61) |
| Alanine aminotransferase, U/L | 1527 | 23.45 (26.16) |
| Albumin, g/dL | 1463 | 3.20 (0.71) |
| Alkaline phosphatase, U/L | 1443 | 277.10 (159.84) |
| Aspartate aminotransferase, U/L | 1527 | 25.60 (27.05) |
| Basophil, U/L | 1303 | 26.55 (57.16) |
| β_2_-microglobulin, mg/dL | 1115 | 6.32 (6.75) |
| Blood urea nitrogen, mg/dL | 1533 | 22.13 (14.32) |
| Calcium, mg/dL | 1462 | 8.76 (0.91) |
| Chloride, mEq/L | 1480 | 104.29 (4.08) |
| Cholinesterase, U/L | 856 | 220.50 (94.44) |
| Creatinine, mg/dL | 1537 | 1.27 (1.32) |
| Eosinophil, U/L | 1395 | 116.89 (152.33) |
| Erythrocyte count, 10^4^/µL | 1530 | 463.53 (2288.16) |
| Gamma-glutamyl transferase, U/L | 1339 | 43.97 (68.64) |
| Hemoglobin, g/dL | 1548 | 9.86 (2.12) |
| Immunoglobulin A, mg/dL | 1359 | 618.57 (1344.43) |
| Immunoglobulin G, mg/dL | 1113 | 2604.63 (2542.80) |
| Immunoglobulin M, mg/dL | 1071 | 56.62 (364.30) |
| Lactate dehydrogenase, U/L | 1523 | 218.40 (278.72) |
| Leukocyte, U/L | 1530 | 5675.72 (5391.33) |
| Lymphocyte, U/L | 1428 | 1388.35 (1020.23) |
| Monocyte, U/L | 1424 | 374.77 (472.92) |
| Platelet, 10^4^/µL | 1530 | 19.27 (18.24) |
| Potassium, mEq/L | 1505 | 4.11 (0.55) |
| Protein fractionation gamma globulin, g/dL | 923 | 1.85 (2.01) |
| Segmented neutrophil, % | 1089 | 61.37 (16.95) |
| Sodium, mEq/L | 1500 | 138.08 (4.00) |
| Total bilirubin, mg/dL | 1498 | 0.51 (0.33) |
| Total cholesterol, mg/dL | 893 | 158.17 (51.82) |
| Total protein, g/dL | 1491 | 7.77 (1.96) |
| Uric acid, mg/dL | 1442 | 5.46 (2.08) |

Abbreviations: ISS = international staging system; SD = standard deviation.

^a^0: no treatment history with drug, or data unavailable; 1: history of treatment of ≥ 1 dose of drug.

^b^0: no laboratory test available; 1: laboratory test available.
