# Supplementary material for: Survival outcomes among patients with multiple myeloma in the era of novel agents: exploratory assessment using an electronic medical record database in Japan
Source: PLoS One. 2023 May 31;18(5):e0285947. doi: 10.1371/journal.pone.0285947 (PMC10231788; doi:10.1371/journal.pone.0285947)
Supplement: S2 Table — (DOCX) [file pone.0285947.s002.docx]

### Table S2. Baseline factors associated with overall survival by Cox proportional hazards model.

| **Variable** | **Variable group** | **Coefficient** | **HR (95% CI)** | ***P* value** |
| --- | --- | --- | --- | --- |
| Rebamipide | Drug | -0.283 | 0.754  (0.578–0.984) | 0.037 |
| Monocyte, U/L | Blood | -0.279 | 0.757  (0.449–1.274) | 0.293 |
| Neutrophil lymphocyte ratio | Blood | -0.279 | 0.757  (0.561–1.020) | 0.067 |
| Lymphocyte, U/L | Blood | -0.264 | 0.768  (0.457–1.292) | 0.318 |
| Erythrocyte, 10^4^/µL | Blood | -0.256 | 0.774  (0.609–0.984) | 0.036 |
| Platelet lymphocyte ratio | blood | -0.232 | 0.793  (0.514–1.223) | 0.293 |
| Albumin, g/dL | Blood | -0.216 | 0.806  (0.701–0.926) | 0.002 |
| Famotidine | Drug | -0.213 | 0.808  (0.619–1.055) | 0.116 |
| Eosinophil, U/L | Blood | -0.212 | 0.809  (0.496–1.318) | 0.392 |
| Chloride, mEq/L | Blood | -0.196 | 0.822  (0.726–0.931) | 0.002 |
| Creatinine, mg/dL | Blood | -0.184 | 0.832  (0.717–0.965) | 0.015 |
| Sennoside | Drug | -0.144 | 0.866  (0.673–1.114) | 0.263 |
| Total protein, g/dL | Blood | -0.128 | 0.880  (0.735–1.054) | 0.164 |
| Immunoglobulin G, mg/dL | Blood | -0.111 | 0.895  (0.739-1.085) | 0.256 |
| Aspirin | Drug | -0.109 | 0.896  (0.699–1.150) | 0.388 |
| Esomeprazole magnesium hydrate | Drug | -0.106 | 0.900  (0.689–1.174) | 0.436 |
| Alanine aminotransferase | Blood | -0.087 | 0.916  (0.813-1.033) | 0.153 |
| Immunoglobulin M, mg/dL | Blood | -0.085 | 0.919  (0.809–1.043) | 0.181 |
| Aspartate aminotransferase, U/L | Blood | -0.081 | 0.922  (0.814–1.045) | 0.204 |
| Glucose (qualitative analysis) | Urine | -0.069 | 0.934  (0.643–1.356) | 0.713 |
| Platelet, 10^4^/µL | Blood | -0.069 | 0.934  (0.715–1.218) | 0.612 |
| Aciclovir | Drug | -0.061 | 0.941  (0.727–1.218) | 0.643 |
| Immunoglobulin A, mg/dL | Blood | -0.056 | 0.946  (0.817–1.094) | 0.449 |
| Alkaline phosphatase, U/L | Blood | -0.048 | 0.953  (0.860–1.056) | 0.356 |
| Total cholesterol, mg/dL | Blood | -0.047 | 0.954  (0.803-–1.133) | 0.585 |
| Cholinesterase, U/L | Blood | -0.043 | 0.958  (0.809–1.134) | 0.607 |
| Segmented neutrophil, % | Blood | -0.029 | 0.971  (0.841–1.122) | 0.688 |
| Protein fractionation gamma globulin, g/dL | Blood | -0.024 | 0.976  (0.864–1.103) | 0.693 |
| Blood urea nitrogen, mg/dL | Blood | -0.024 | 0.976  (0.854–1.116) | 0.724 |
| Fluconazole | Drug | -0.023 | 0.977  (0.771–1.238) | 0.849 |
| Total bilirubin, mg/dL | Blood | -0.016 | 0.984  (0.881–1.099) | 0.773 |
| Sodium, mEq/L | Blood | 0.011 | 1.011  (0.871–1.174) | 0.883 |
| Acetaminophen | Drug | 0.016 | 1.016  (0.766–1.347) | 0.913 |
| Basophil lymphocyte ratio | Blood | 0.018 | 1.018  (0.585–1.773) | 0.949 |
| Bortezomib + lenalidomide | 1^st^ Regimen | 0.019 | 1.020  (0.549–1.893) | 0.951 |
| Protein (qualitative analysis) | Urine | 0.020 | 1.020  (0.795–1.310) | 0.872 |
| Age 65–74 years | Age | 0.026 | 1.026  (0.730–1.442) | 0.881 |
| Basophil, U/L | Blood | 0.032 | 1.032  (0.576–1.851) | 0.913 |
| Stage II | ISS | 0.034 | 1.035  (0.757–1.413) | 0.830 |
| Uric acid, mg/dL | Blood | 0.036 | 1.037  (0.931–1.154) | 0.510 |
| Lansoprazole | Drug | 0.042 | 1.043  (0.848–1.283) | 0.689 |
| Potassium, mEq/L | Blood | 0.048 | 1.050  (0.955–1.153) | 0.313 |
| Magnesium oxide | Drug | 0.057 | 1.059  (0.872–1.287) | 0.562 |
| Gamma-glutamyl transferase, U/L | Blood | 0.067 | 1.069  (0.948–1.206) | 0.273 |
| C-reactive protein, mg/dL | Blood | 0.070 | 1.072  (0.974–1.181) | 0.153 |
| Febuxostat | Drug | 0.081 | 1.085  (0.836–1.408) | 0.541 |
| Hemoglobin, g/dL | Blood | 0.092 | 1.096  (0.937–1.282) | 0.252 |
| Amlodipine besylate | Drug | 0.095 | 1.100  (0.863–1.403) | 0.441 |
| Furosemide | Drug | 0.102 | 1.107  (0.866–1.415) | 0.416 |
| Isotonic sodium chloride solution | Drug | 0.108 | 1.114  (0.866–1.415) | 0.372 |
| Other | 1^st^ regimen | 0.115 | 1.122  (0.872–1.443) | 0.371 |
| β_2_-microglobulin, mg/dL | Blood | 0.115 | 1.122  (0.917–1.373) | 0.258 |
| Eosinophil lymphocyte ratio | Blood | 0.122 | 1.129  (0.706–1.807) | 0.610 |
| Calcium, mg/dL | Blood | 0.123 | 1.131  (1.008–1.268) | 0.036 |
| Urobilinogen, qualitative analysis | Urine | 0.126 | 1.134  (0.765–1.680) | 0.521 |
| Male | Sex | 0.145 | 1.156  (0.956–1.398) | 0.135 |
| > 74, years | Age | 0.164 | 1.178  (0.695–1.997) | 0.542 |
| Sulfamethoxazole trimethoprim | Drug | 0.186 | 1.205  (0.976–1.487) | 0.083 |
| Monocyte lymphocyte ratio | Blood | 0.225 | 1.252  (0.716–2.189) | 0.429 |
| Age, years | Age | 0.225 | 1.253  (1.013–1.549) | 0.037 |
| Stage III | ISS | 0.284 | 1.329  (0.860–2.053) | 0.198 |
| Lenalidomide | First regimen | 0.343 | 1.410  (1.056–1.883) | 0.020 |
| Leukocyte, U/L | Blood | 0.361 | 1.434  (1.083–1.899) | 0.012 |
| Lactate dehydrogenase, U/L | Blood | 0.400 | 1.492  (1.321-–1.685) | 0.000 |

Abbreviations: CI = confidence interval; HR = hazard ratio; ISS = international staging system.
