# Supplementary material for: Survival outcomes among patients with multiple myeloma in the era of novel agents: exploratory assessment using an electronic medical record database in Japan
Source: PLoS One. 2023 May 31;18(5):e0285947. doi: 10.1371/journal.pone.0285947 (PMC10231788; doi:10.1371/journal.pone.0285947)
Supplement: S3 Table — (DOCX) [file pone.0285947.s003.docx]

### Table S3. Baseline factors associated with overall survival by random survival forest model.

| **Variable** | **Variable group** | **Permutation importance** |
| --- | --- | --- |
| Lactate dehydrogenase, U/L | Blood | 0.0053 |
| β_2_-microglobulin, mg/dL | Blood | 0.0051 |
| Platelet, 10^4^/µL | Blood | 0.0039 |
| ISS | Demographic | 0.0033 |
| Age, years | Demographic | 0.0029 |
| Erythrocyte, 10^4^/µL | Blood | 0.0028 |
| Albumin, g/dL | Blood | 0.0026 |
| Hemoglobin, g/dL | Blood | 0.0025 |
| Chloride, mEq/L | Blood | 0.0020 |
| Blood urea nitrogen, mg/dL | Blood | 0.0020 |
| Neutrophil lymphocyte ratio | Blood | 0.0018 |
| Age group, years | Demographic | 0.0017 |
| Cholinesterase, U/L | Blood | 0.0016 |
| Total protein, g/dL | Blood | 0.0015 |
| C-reactive protein, mg/dL | Blood | 0.0014 |
| Lymphocyte, U/L | Blood | 0.0013 |
| Gamma-glutamyl transferase, U/L | Blood | 0.0013 |
| Platelet lymphocyte ratio | Blood | 0.0012 |
| Total cholesterol, mg/dL | Blood | 0.0010 |
| Monocyte lymphocyte ratio | Blood | 0.0010 |
| Eosinophil, U/L | Blood | 0.0009 |
| Creatinine, mg/dL | Blood | 0.0008 |
| Segmented neutrophil | Blood | 0.0007 |
| Immunoglobulin M, mg/dL | Blood | 0.0006 |
| Sodium, mEq/L | Blood | 0.0005 |
| Furosemide | Drug | 0.0005 |
| Uric acid, mg/dL | Blood | 0.0005 |
| Potassium, mEq/L | Blood | 0.0005 |
| Eosinophil lymphocyte ratio | Blood | 0.0005 |
| Aspartate aminotransferase, U/L | Blood | 0.0003 |
| Immunoglobulin G, mg/dL | Blood | 0.0003 |
| Sex | Demographic | 0.0002 |
| Calcium, mg/dL | Blood | 0.0002 |
| Leukocyte, U/L | Blood | 0.0002 |
| Monocyte, U/L | Blood | 0.0002 |
| Protein (qualitative analysis) | Urine | 0.0002 |
| Rebamipide | Drug | 0.0002 |
| Alkaline phosphatase, U/L | Blood | 0.0002 |
| Alanine aminotransferase, U/L | Blood | 0.0002 |
| Protein fractionation gamma globulin, g/dL | Blood | 0.0001 |
| Basophil lymphocyte ratio | Blood | 0.0001 |
| Esomeprazole magnesium hydrate | Drug | 0.0001 |
| Total bilirubin, mg/dL | Blood | 0.0001 |
| Sulfamethoxazole trimethoprim | Drug | 0.0001 |
| Febuxostat | Drug | 0.0000 |
| Regimen | First regimen | 0.0000 |
| Fluconazole | Drug | 0.0000 |
| Glucose (qualitative analysis) | Urine | 0.0000 |
| Amlodipine besylate | Drug | 0.0000 |
| Isotonic sodium chloride solution | Drug | 0.0000 |
| Famotidine | Drug | 0.0000 |
| Sennoside | Drug | 0.0000 |
| Acetaminophen | Drug | 0.0000 |
| Urobilinogen (qualitative analysis) | Urine | 0.0000 |
| Lansoprazole | Drug | 0.0000 |
| Basophil, U/L | Blood | 0.0000 |
| Aciclovir | Drug | 0.0000 |
| Aspirin | Drug | 0.0000 |
| Immunoglobulin A, mg/dL | Blood | -0.0001 |
| Magnesium oxide | Drug | -0.0001 |

Abbreviation: ISS = international staging system.
