# Supplementary material for: Survival outcomes among patients with multiple myeloma in the era of novel agents: exploratory assessment using an electronic medical record database in Japan
Source: PLoS One. 2023 May 31;18(5):e0285947. doi: 10.1371/journal.pone.0285947 (PMC10231788; doi:10.1371/journal.pone.0285947)
Supplement: S4 Table — (DOCX) [file pone.0285947.s004.docx]

### Table S4. Univariate analysis of overall survival by continuous variables.

| **Variable** | **Variable group** | **Cutoff value^a^** | **HR**  **(95% CI)** | ***P* value** |
| --- | --- | --- | --- | --- |
| Platelet, 10^4^/µL | Blood | 8.2 | 2.52  (2.04–3.11) | < 0.0001 |
| Immunoglobulin E nonspecific, U/mL | Blood | 29 | 2.00  (1.01–3.97) | 0.04784 |
| Platelet lymphocyte ratio | Blood | 0.00606 | 1.88  (1.49–2.37) | < 0.0001 |
| Hematocrit, % | Blood | 26.2 | 1.86  (1.59–2.18) | < 0.0001 |
| Erythrocyte, 10^4^/µL | Blood | 298 | 1.85  (1.59–2.16) | < 0.0001 |
| Hemoglobin, g/dL | Blood | 9.55 | 1.80  (1.55–2.1) | < 0.0001 |
| Triglyceride, mg/dL | Blood | 51 | 1.80  (1.26–2.56) | 0.00127 |
| Zinc sulfate turbidity test, U/L | Blood | 1.25 | 1.79  (1.37–2.32) | < 0.0001 |
| Cholinesterase, U/L | Blood | 186 | 1.75  (1.43–2.14) | < 0.0001 |
| Albumin, g/dL | Blood | 2.95 | 1.71  (1.46–2.00) | < 0.0001 |
| Low-density lipoprotein cholesterol, mg/dL | Blood | 46 | 1.68  (1.15–2.46) | 0.00792 |
| Creatinine, mg/dL | Urine | 109 | 1.62  (1.09–2.39) | 0.0162 |
| Magnesium, mg/dL | Blood | 1.8 | 1.52  (1.02–2.26) | 0.0403 |
| Total cholesterol, mg/dL | Blood | 162 | 1.50  (1.23–1.83) | < 0.0001 |
| Immunoglobulin G, mg/dL | Blood | 591 | 1.49  (1.24–1.79) | < 0.0001 |
| Calcium, mg/dL | Blood | 7.86 | 1.48  (1.21–1.83) | 0.000192 |
| Eosinophil lymphocyte ratio | Blood | 0.00174 | 1.48  (1.18–1.86) | 0.000795 |
| Chloride, mEq/L | Blood | 101 | 1.47  (1.24–1.76) | < 0.0001 |
| Total protein, g/dL | Blood | 6.4 | 1.47  (1.24–1.74) | < 0.0001 |
| Immunoglobulin M, mg/dL | Blood | 19.4 | 1.45  (1.21–1.74) | < 0.0001 |
| Lymphocyte, µL | Blood | 1215 | 1.45  (1.24–1.70) | < 0.0001 |
| Eosinophil, µL | Blood | 44 | 1.44  (1.23–1.70) | < 0.0001 |
| Potassium, mEq/L | Blood | 3.89 | 1.37  (1.17–1.61) | 0.000108 |
| Inorganic phosphate, mg/dL | Blood | 2.45 | 1.33  (0.97–1.83) | 0.0784 |
| Basophil, µL | Blood | 0.0391 | 1.32  (1.10–1.59) | 0.00243 |
| Basophil lymphocyte ratio | Blood | 0 | 1.32  (1.10–1.58) | 0.00264 |
| Protein fractionation gamma globulin, g/dL | Blood | 3.93 | 1.32  (1.01–1.73) | 0.0437 |
| High-density lipoprotein cholesterol, mg/dL | Blood | 43 | 1.26  (0.96–1.66) | 0.094 |
| Monocyte, µL | Blood | 172 | 1.26  (1.03–1.53) | 0.0235 |
| Sodium, mEq/L | Blood | 138 | 1.23  (1.05–1.43) | 0.00814 |
| Alanine aminotransferase, u/L | Blood | 21.4 | 1.09  (0.93–1.28) | 0.286 |
| Segmented neutrophil, % | Blood | 65.6 | 0.84  (0.70–1.00) | 0.0474 |
| Immunoglobulin A, mg/dL | Blood | 427 | 0.78  (0.65–0.94) | 0.00832 |
| Alkaline phosphatase, U/L | Blood | 429 | 0.77  (0.61–0.97) | 0.0294 |
| Total bilirubin, mg/dL | Blood | 0.72 | 0.77  (0.62–0.96) | 0.0226 |
| Uric acid, mg/dL | Blood | 5.78 | 0.77  (0.66–0.9) | 0.000841 |
| Band neutrophil, % | Blood | 3.67 | 0.74  (0.58–0.94) | 0.014 |
| Monocyte lymphocyte ratio | Blood | 0.214 | 0.74  (0.63–0.87) | 0.000271 |
| Gamma-glutamyl transferase, U/L | Blood | 37 | 0.72  (0.60–0.85) | 0.000156 |
| Neutrophil, µL | Blood | 5934 | 0.68  (0.53–0.86) | 0.00161 |
| Leukocyte, µL | Blood | 8850 | 0.67  (0.53–0.85) | 0.000988 |
| C-reactive protein, mg/dL | Blood | 0.667 | 0.65  (0.55–0.77) | < 0.0001 |
| Creatinine, mg/dL | Blood | 1.02 | 0.65  (0.55–0.76) | < 0.0001 |
| Estimated glomerular filtration rate by creatinine, mL/min | Blood | 127 | 0.65  (0.56–0.76) | < 0.0001 |
| Protein quantitative to creatinine ratio, g/g(creat) | Urine | 0.94 | 0.64  (0.47–0.87) | 0.00405 |
| Aspartate aminotransferase, U/L | Blood | 38 | 0.63  (0.50–0.79) | < 0.0001 |
| Protein quantitative, mg/dL | Urine | 15.1 | 0.63  (0.46-–0.86) | 0.00393 |
| Immunoglobulin D, mg/dL | Blood | 1 | 0.60  (0.29–1.21) | 0.153 |
| Neutrophil lymphocyte ratio | Blood | 4.92 | 0.60  (0.48–0.73) | < 0.0001 |
| Age, years | Demographic | 71 | 0.58  (0.50–0.68) | < 0.0001 |
| Blood urea nitrogen, mg/dL | Blood | 23.9 | 0.58  (0.49–0.68) | < 0.0001 |
| Lactate dehydrogenase, U/L | Blood | 266 | 0.48  (0.39–0.58) | < 0.0001 |
| β_2_-microglobulin, mg/L | Blood | 4.88 | 0.45  (0.38–0.54) | < 0.0001 |

Abbreviations: CI = confidence interval; HR = hazard ratio.

^a^Value shown is the upper limit of the cutoff value.
