# Supplementary material for: Survival outcomes among patients with multiple myeloma in the era of novel agents: exploratory assessment using an electronic medical record database in Japan
Source: PLoS One. 2023 May 31;18(5):e0285947. doi: 10.1371/journal.pone.0285947 (PMC10231788; doi:10.1371/journal.pone.0285947)
Supplement: S5 Table — (DOCX) [file pone.0285947.s005.docx]

### Table S5. Accuracy of each prognostic classification model constructed using a decision tree.

| **Rule** | **C-Index** |
| --- | --- |
| Decision Tree 1 | 0.643 |
| Decision Tree 2 | 0.614 |
| Decision Tree 3 | 0.629 |
| Decision Tree 4 | 0.641 |
| International Staging System | 0.638 |
