# Supplementary material for: Survival outcomes among patients with multiple myeloma in the era of novel agents: exploratory assessment using an electronic medical record database in Japan
Source: PLoS One. 2023 May 31;18(5):e0285947. doi: 10.1371/journal.pone.0285947 (PMC10231788; doi:10.1371/journal.pone.0285947)
Supplement: S1 Fig — (DOCX) [file pone.0285947.s006.docx]

### Fig S1. Survival outcomes stratified with the decision tree.


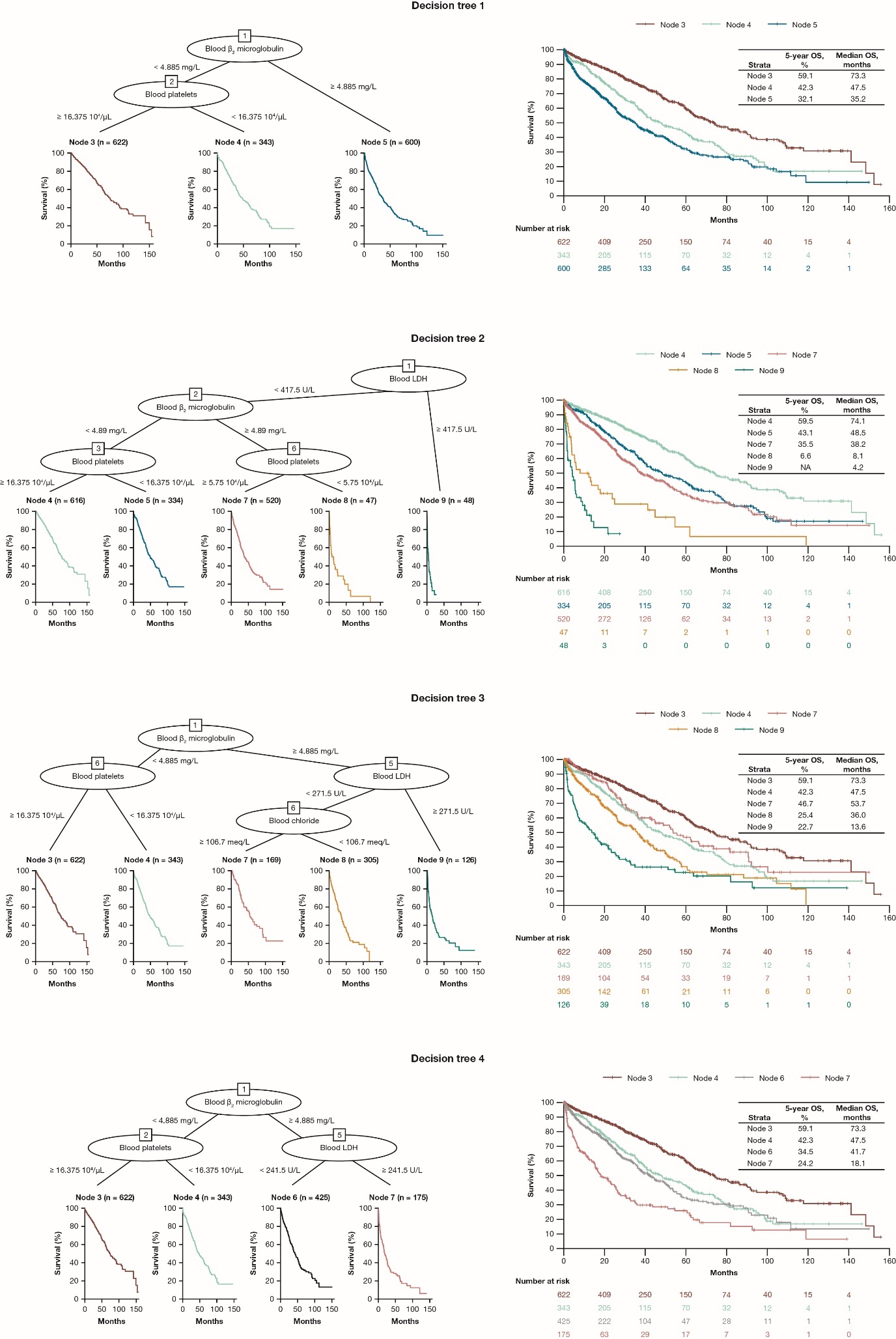


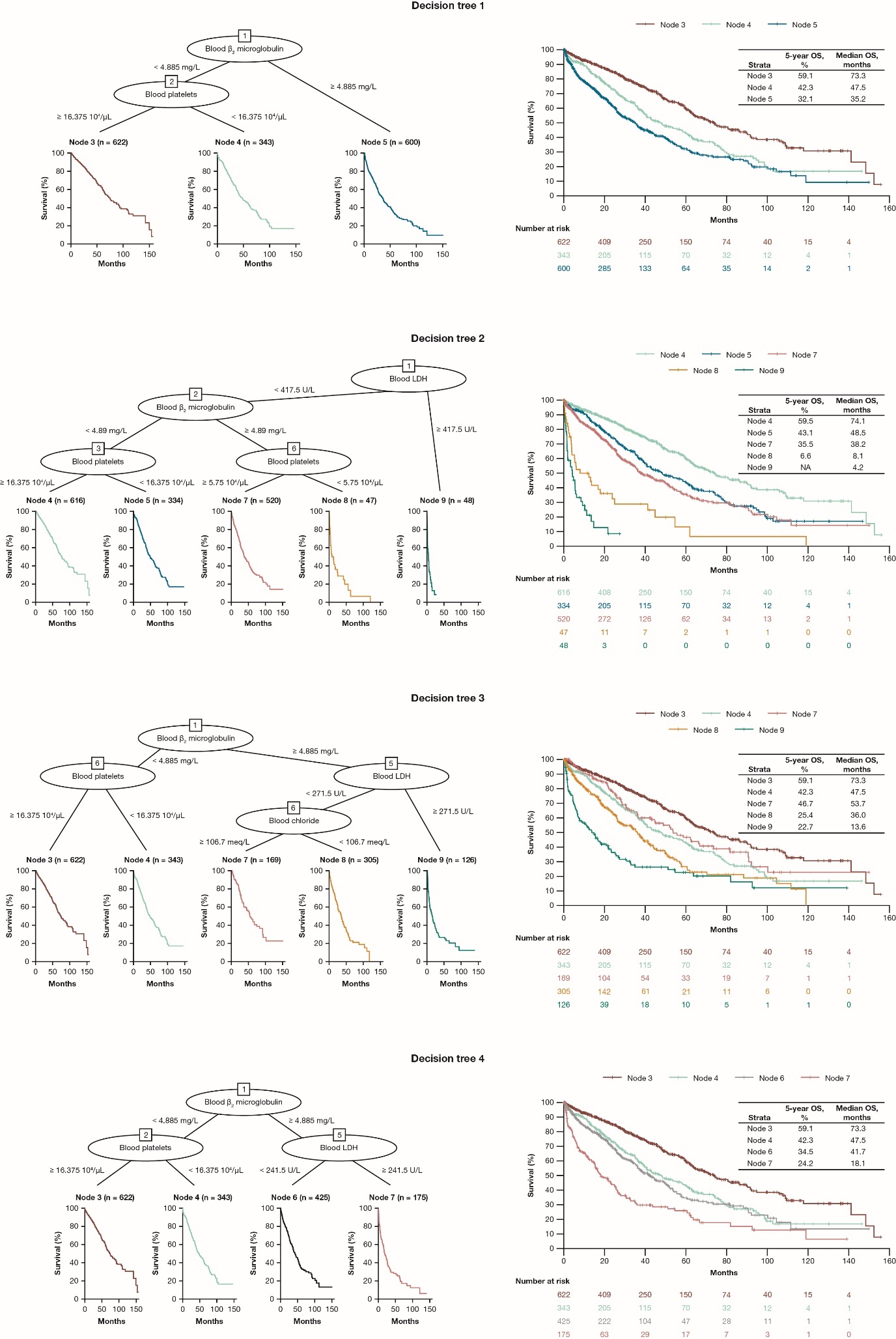

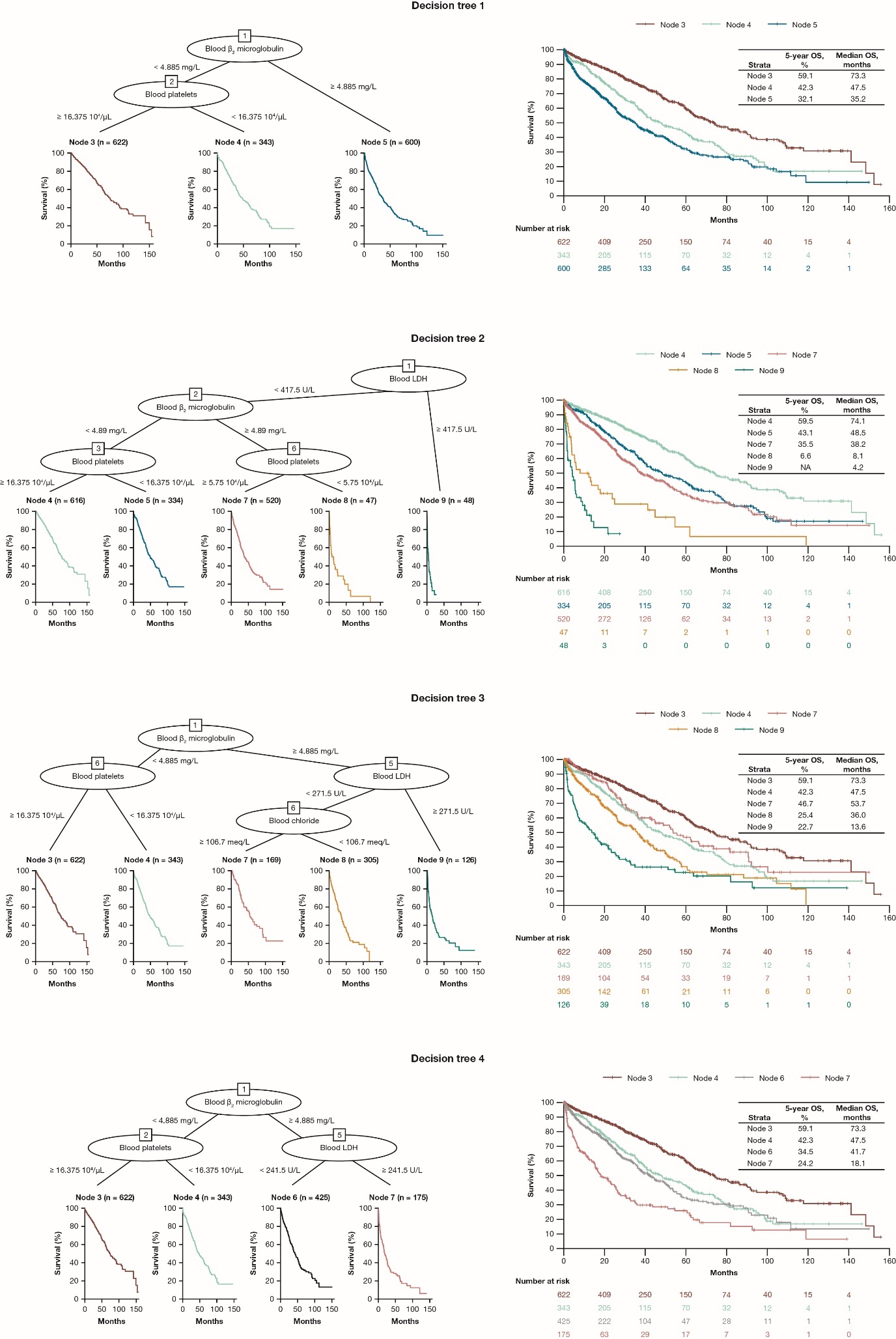

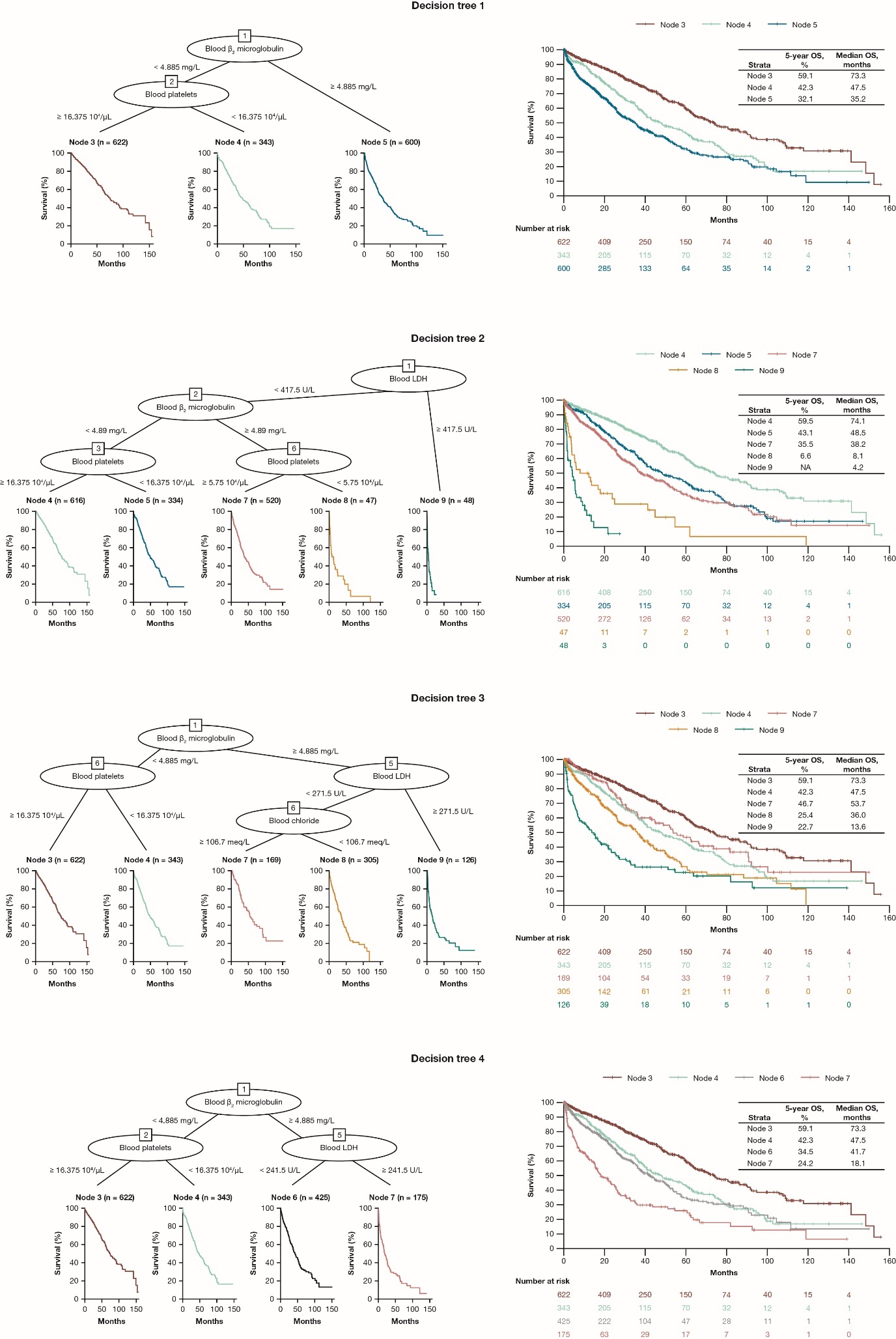


The hyperparameters with a grid search used in the random survival forest model were as follows: trees, n = 450; variables used in each tree, n = 3; minimum leaf node size, 3; tree depth, unlimited; tree split criteria, C-Index. Cross-validation (8-fold) was performed on both the Cox proportional hazards model and the random survival forest model to evaluate the model accuracy both in CV-train and CV-test.

Abbreviations: CV-test = cross-validation in testing data; CV-train = cross-validation in training data; LDH = lactate dehydrogenase; OS = overall survival.
